# tachAId—An interactive tool supporting the design of human-centered AI solutions

**DOI:** 10.3389/frai.2024.1354114

**Published:** 2024-03-12

**Authors:** Max Bauroth, Pavlos Rath-Manakidis, Valentin Langholf, Laurenz Wiskott, Tobias Glasmachers

**Affiliations:** ^1^Faculty of Computer Science, Institute for Neural Computation, Ruhr University Bochum, Bochum, Germany; ^2^Institute of Work Science, Ruhr University Bochum, Bochum, Germany

**Keywords:** tachAId, artificial intelligence, human-centered AI, human-centered design, human-centered design goals, human-AI interaction (HAII)

## Abstract

In an era where Artificial Intelligence (AI) integration into business processes is crucial for maintaining competitiveness, there is a growing need for structured guidance on designing AI solutions that align with human needs. To this end, we present “technical assistance concerning human-centered AI development” (tachAId), an interactive advisory tool which comprehensively guides AI developers and decision makers in navigating the machine learning lifecycle with a focus on human-centered design. tachAId motivates and presents concrete technical advice to ensure human-centeredness across the phases of AI development. The tool's effectiveness is evaluated through a catalog of criteria for human-centered AI in the form of relevant challenges and goals, derived from existing methodologies and guidelines. Lastly, tachAId and one other comparable advisory tool were examined to determine their adherence to these criteria in order to provide an overview of the human-centered aspects covered by these tools and to allow interested parties to quickly assess whether the tools meet their needs.

## 1 Introduction

Increasingly complex and powerful technologies are being developed that rapidly change our world. According to people like Bill Gates (Gates, 2023) the most important technological development in recent years and the foreseeable future is Artificial Intelligence (AI). This is reflected in the growth of the AI market, which has been roughly $95.6 billion in 2021, $142.3 billion in 2022 and is expected to reach $1,847.5 billion by 2030 (Statista, 2023). Based on these facts alone, it is safe to say that AI is here to stay. Diverse AI applications are already involved in a range of everyday tasks and activities. However, most of these interactions take place outside of work or their effect on work outputs and conditions are not systematically assessed, let alone organized.

Given that we spend on average between 20% and 25% of our wakeful lives at work (Campbell, 2017; Office for National Statistics, 2023), it is imperative to systematically study and learn to anticipate the impact of AI on work processes. In addition, studies have shown that the demand for AI in companies is increasing. On average, 35% of companies are already using AI, and an additional 42% are exploring its use as of 2022 (IBM, 2022b). That is an overall increase of 4% over the last 2 years while a total of 77% of companies are already dealing with AI in some capacity.

This transition comes with noticeable effects. Industries as diverse as finance, healthcare, transportation, biotechnology, or gaming, to name but a few, are adopting AI solutions (Lee et al., 2022). And just as diverse as the sectors are the professions working with this technology. AI is long past the stage where it has been the concern of software engineers alone. In fact, it is expected to disrupt, create, and redefine hundreds of millions of jobs permanently changing the way people work (Di Battista et al., 2023; Hatzius et al., 2023).

All of the above shows that it is particularly important to study AI in a work context; and since in many cases AI is likely to work with or affect humans in some way, it is of particular interest to study how it interacts with them, and to ensure that these interactions are not detrimental to the affected individuals. This particular area of research is called human-centered AI (HCAI). It is an emerging discipline aimed at creating AI systems that collaborate with humans, rather than compete with or replace them. The focus here is on considering the human from the beginning and throughout the whole design process.

There are numerous examples of AI solutions that were not designed with all of the potential impacts on humans in mind. As a result, AI has performed worse than humans alone, or in some cases has even had harmful effects on people. For instance, in 2018 it was made public that Amazon had been developing an AI to partially automate its hiring process based on the resumes of prospective employees. However, the AI did not perform as planned and ended up showing a discriminatory bias against women (Dastin, 2018). At the same time, there are numerous examples that underline how AI solutions that are properly designed for human needs and wishes can benefit not only the people who work with them, but also their companies in general. For instance, in finance, it is being successfully used to detect fraudulent credit card behavior. Suspicious transactions are flagged for human evaluation, while trustworthy transactions are handled automatically, freeing the investigators to focus on important cases (Wilson and Daugherty, 2018).

Much of the literature suggests that the problem of HCAI can be represented in its entirety by three distinct topics: the technical aspect, the ethical or humanistic aspect, and the interplay between them through technically-mediated interactions. For instance, Auernhammer (2020) proposes a framework comprising (1) the human, (2) policies and guidelines, and (3) technology, which he terms as humanistic design, judicial and rationalistic aspects, respectively. Within this framework, the judicial aspect, which is closely related to ethics, serves to inform, assess, and refine technology and its impact on humans as well as its use, thereby shaping emerging human-technology symbioses.

Other researchers directly incorporate ethical principles into their frameworks and position ethics as a key factor therein. Degen and Ntoa (2021) and Xu (2019) propose such HCAI frameworks. The framework by Degen and Ntoa (2021) builds upon the three major research topics they identified as relevant for HCAI: AI and trust, the technical AI system, and the human-centered AI process. Xu (2019) proposes an extended HCAI framework that comprises three main components. First, the ethically aligned design, which focuses on creating AI solutions that do not discriminate against humans, act fairly and justly and do not replace humans. Second, technological enhancement, and, finally, the human factors design, to ensure that the proposed AI solutions are useful, usable, and can be thoroughly inspected and understood.

In all three frameworks, ethics provide a guideline, a motivation, or a kind of supervision to which the design of the AI should adhere.

Examples of general guidelines on HCAI include Amershi et al. (2019) who synthesized guidelines out of a vast literature search, primarily concerned with human-AI interaction and Microsoft (2022) who released a guide on creating responsible AI. By and large, they identified six major goals: (1) accountability, (2) transparency, (3) fairness, (4) reliability and safety, (5) privacy and security and finally, (6) inclusiveness. All of which are subdivided into more detailed goals and specific requirements for their realization.

At the policy level, the European Union has published guidelines on ethics for trustworthy AI (EU, 2019). The authors derived seven core principles from an ethical point of view: (1) human agency and oversight, (2) technical robustness and safety, (3) privacy and data governance, (4) transparency, (5) diversity, non-discrimination and fairness, (6) societal and environmental well-being and (7) accountability. Hickman and Petrin (2021) have studied the EU's ethical guidelines from a company law perspective and have pointed out that although being ethically accurate, the guidelines often fail to go into great detail or simply neglect specific topics. They therefore fail to immediately aid in HCAI design.

Moving beyond general frameworks that set the stage for human-centered AI design and help structure it as a process Hartikainen et al. (2023) and Bingley et al. (2023), who argue that although general frameworks help validate, may help to inspire or inform, they ultimately fail to aid in building HCAI.

Wilkens et al. (2021) contributed a taxonomy of perspectives specific to AI in the workplace. They reviewed the literature and synthesized five co-existing understandings of human-centricity in the workplace:

1. a **deficit-oriented** understanding that addresses the use of AI in order to ease the burden on the work force in terms of attention, fatigue, etc.
2. a **data reliability-oriented** understanding that is about explainability and trustworthiness in the context of fairness and unbiased data.
3. a **protection-oriented** understanding that focuses on human-centered design, ergonomics, and physical and mental well-being when working with AI.
4. a **potential-oriented** understanding that stresses the fact that humans and AI shall work together by leveraging the potentials of human productivity through AI.
5. a **political-oriented** understanding of how to reach human centricity while using AI in the workplace about the normative criterion of AI subordination under human interests.

These perspectives, i.e., understandings, help to identify and holistically understand challenges and goals that have to be considered for applications of HCAI in the workplace.

In light of the aforementioned challenges, there is a need for specific rules grounded in relevant ethical principles complete with guidance and advice in terms of incorporating or rather designing AI solutions specifically adjusted to human needs in a work environment. This paper addresses this need by proposing tachAId, an interactive tool, designed to aid company stakeholders and AI developers to design human-centered AI solutions. The name tachAId stands for technical assistance concerning human-centered AI development. tachAId guides along the phases of AI development, points at potential challenges at the points of contact between humans and AI, and maps these challenges to technical measures and tools; e.g., in the form of algorithms or libraries, that can be used to satisfy diverse requirements toward HCAI, such as not to constrain but empower AI development in a human-centered direction.

In the rest of the paper, we will present our tool and explain its characteristics in the methods section. Moreover, we will also present a validation catalog, synthesized from the presented frameworks, guidelines, and the specific criteria contained therein—extended by the labor science understandings by Wilkens et al. (2021)—, which will help us to assess whether tachAId is suited to provide design advice for human-centered AI solutions. In the results section, we will analyze tachAId along with one other tool, that also has the explicit goal of guiding human-centered AI design, to see which criteria from the validation catalog are met. In the discussion, we will show in detail how tachAId specifically addresses the challenges and goals formulated in the catalog. We will also highlight issues that are currently not yet addressed in our tool. Finally, based on the results and insights gained from the analysis of tachAId with the help of the proposed validation catalog, we will give an outlook on how we want to develop our tool in the future, in order to make tachAId provide users with holistic guidance for the design of their human-centered AI solutions at the workplace.

## 2 Methods

In this section we present the structure and the format of the proposed interactive guide tachAId. Further, we introduce the methodology with which we have derived and organized the specific challenges and goals relevant for the technical realization of HCAI. A special focus lies on incorporating the relevance of the work context and clarifying the interplay between the different disciplines that are involved in HCAI. The identified challenges and goals are used to derive a validation catalog, which we use to validate the comprehensiveness of our and other HCAI guides in the subsequent sections of this paper.

### 2.1 tachAId—the interactive guide

tachAId^1^ (Bauroth and Rath-Manakidis, 2023), the current version of which is dated from 07.12.2023, is an interactive guide that provides concrete recommendations for implementing AI software and capabilities in a human-centered way. The language of the tool is German. The users of tachAId should be able to identify the areas in which stakeholders can contribute to human-centered AI development, how to design the AI system for successful human collaboration, and how such collaboration may be structured. tachAId addresses the challenges of human-AI collaboration and ethical design of AI, and proposes technical solutions that address these challenges.

**Figure 1:**
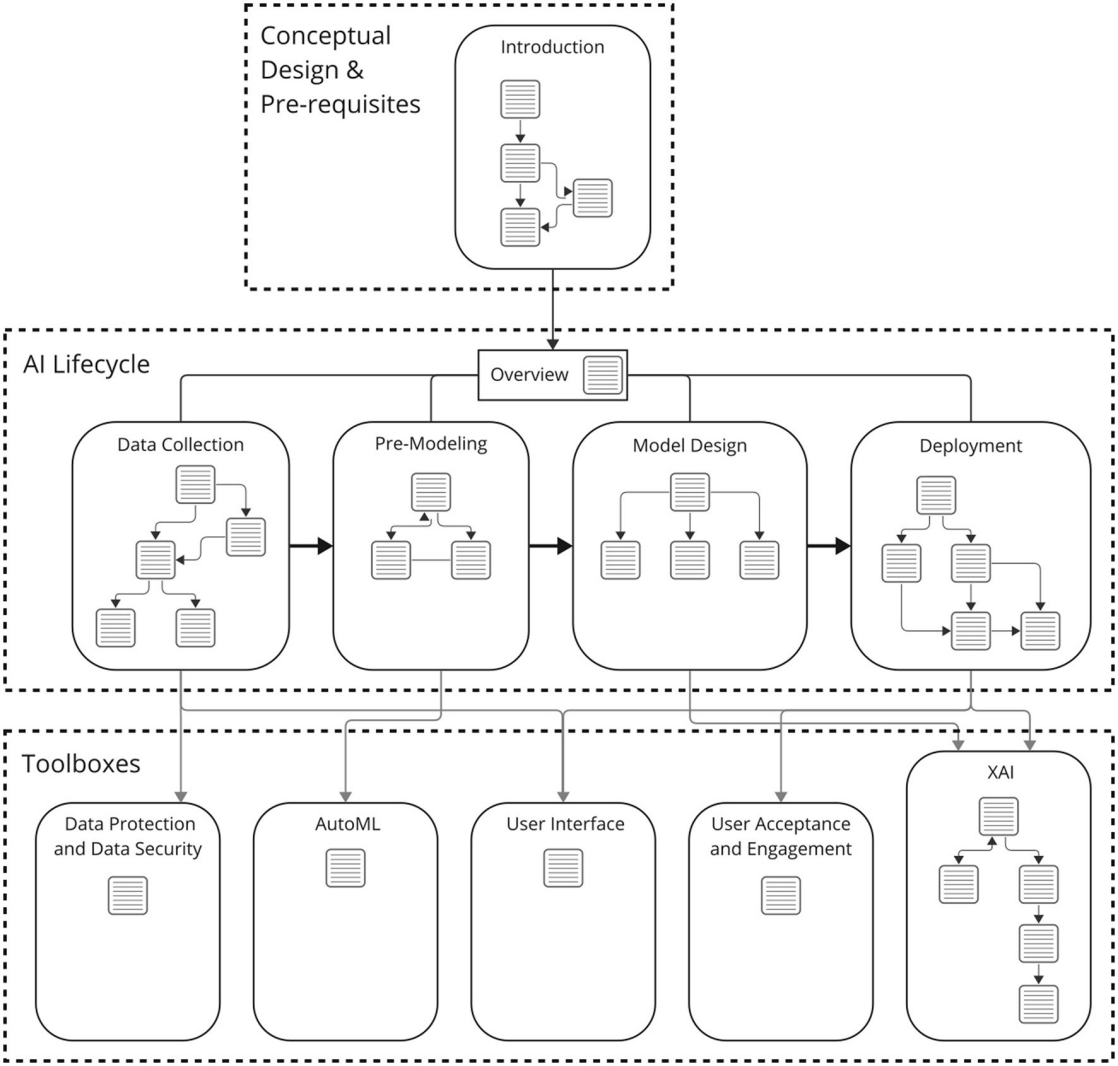
Structure of tachAId and user flow, i.e., navigation, through the tool. The rounded boxes represent different units of content, structured either along the phases of the AI lifecycle (**middle row**), general conceptual pre-development considerations for HCAI (**top**), or task-specific toolboxes (**bottom row**). This content is divided into views, or “slides”, represented by text boxes that the user can explore. Paths between slides are indicated by arrows. The figure shows an excerpt from the December 2023 development version of tachAId.

tachAId is designed as a process-accompanying tool, offering specific recommendations for various stages of technical implementation in AI/ML applications in a work context. It is implemented as an interactive HTML document compatible with web browsers. The structure of tachAId proceeds along the phases of the technical implementation of AI and ML applications, outlined in the CRISP-DM model (Shearer, 2000). The structure of tachAId, i.e., the user journey through the tool, is outlined in Figure 1 and extends from the data collection up to the deployment of the finished model. Furthermore, various toolboxes have been designed to emphasize different core themes in the context of human centered AI design. These are: how to design and implement user interfaces, how to increase user acceptance and engagement, how to secure data privacy and data security, how to design AI that is explainable (XAI), and how to facilitate the overall technical process of designing an AI easier through automated machine learning (AutoML).

The tool has three primary target groups. First, decision makers responsible for the introduction or redesign of AI components in labor processes following a human-centered approach; second organizational stakeholders who seek to support AI-driven transformations by prioritizing human needs; and, thirdly, AI developers who want to be empowered by the knowledge and tooling for the realization of human-centered AI.

#### 2.1.1 Interactive story format

We built tachAId on the twine interactive storytelling platform (Klimas, 2009): The content in tachAId is not organized in a linear manner. Instead, it is progressively and selectively presented based on and customized according to the choices the user makes while interacting with tachAId.

We chose the format of interactive storytelling, because our goal is to provide an engaging, dynamic, and playful way for stakeholders interested in AI that supports the work in their organization to go through the different stages of technical AI implementation and explore the many challenges that arise at the intersection of AI technology and work organization. tachAId poses use-case specific technical and organizational questions that guide the understanding and concretization of human-centered AI. We give the user the agency to explore different stages of AI development and inform them such that they can explore and find answers to common questions, gain awareness and oversight of the AI implementation and deployment process ahead, and have additional content presented to them selectively and self-determinedly based on the insights they gain. Once awareness of concrete needs is established in tachAId, we point to external resources that discuss specific challenges and technical measures, such as tools developed in the context of the humAIne competence center or third-party guidelines and software packages.

The twine platform which underlies tachAId is primarily used for interactive fiction. Interactive fiction is a literary genre and artistic medium wherein the author and UX designer guides the user through a story that unfolds incrementally based on the user's decisions.

Similarly, because of the interactive format of tachAId, users of the tool are not presented with an exhaustive list of possible human-AI teaming challenges that may not exist in the context of their specific application or technical measures that may be inappropriate for the task, the architecture of the underlying AI system, or the mode of user-AI interaction. Instead, interaction is used to prevent fatigue by presenting relevant challenges and possible solutions in a step-by-step fashion, so that takeaways can be drawn quickly and a sense of reward and curiosity can arise. By placing the user in this position of agency to simulate a hypothetical HCAI development process, we want to encourage reflection on the organizational conditions and requirements for successful human-centered AI implementation, as well as judgment about which contents of tachAId are relevant and which are not.

#### 2.1.2 Design, flow, and layout of tachAId

tachAId adopts a clear and simple design with a limited use of different elements and colors. Superfluous information is deliberately minimized, and detailed data is only presented upon user request.

The user journey begins with an initial set of broad conceptual questions of an organizational nature that aim to create an awareness of human-centered design, establish an understanding of the company's mindset, and drive its readiness and willingness to embrace AI in a human-centered way. These foundational questions are critical because they set the stage for the subsequent exploration of specific AI development steps and uncover opportunities and organizational synergies for human-centered AI design. We consider these early inquiries to be a prerequisite for the subsequent technical implementation of the human-centered AI system. The flow of the rest of tachAId generally follows the established AI design process outlined in the CRISP-DM (Cross-Industry Standard Process for Data Mining) methodology. The content is divided into (1) data consolidation and collection, (2) pre-modeling, (3) model design and validation, and (4) deployment. Figure 1 shows the general structure through which the user can progress through the content of the tool. We chose this structure to leverage the interactive format to foster a comprehensive awareness of various potential pitfalls and challenges of (HC)AI systems with the user.

**Figure 2:**
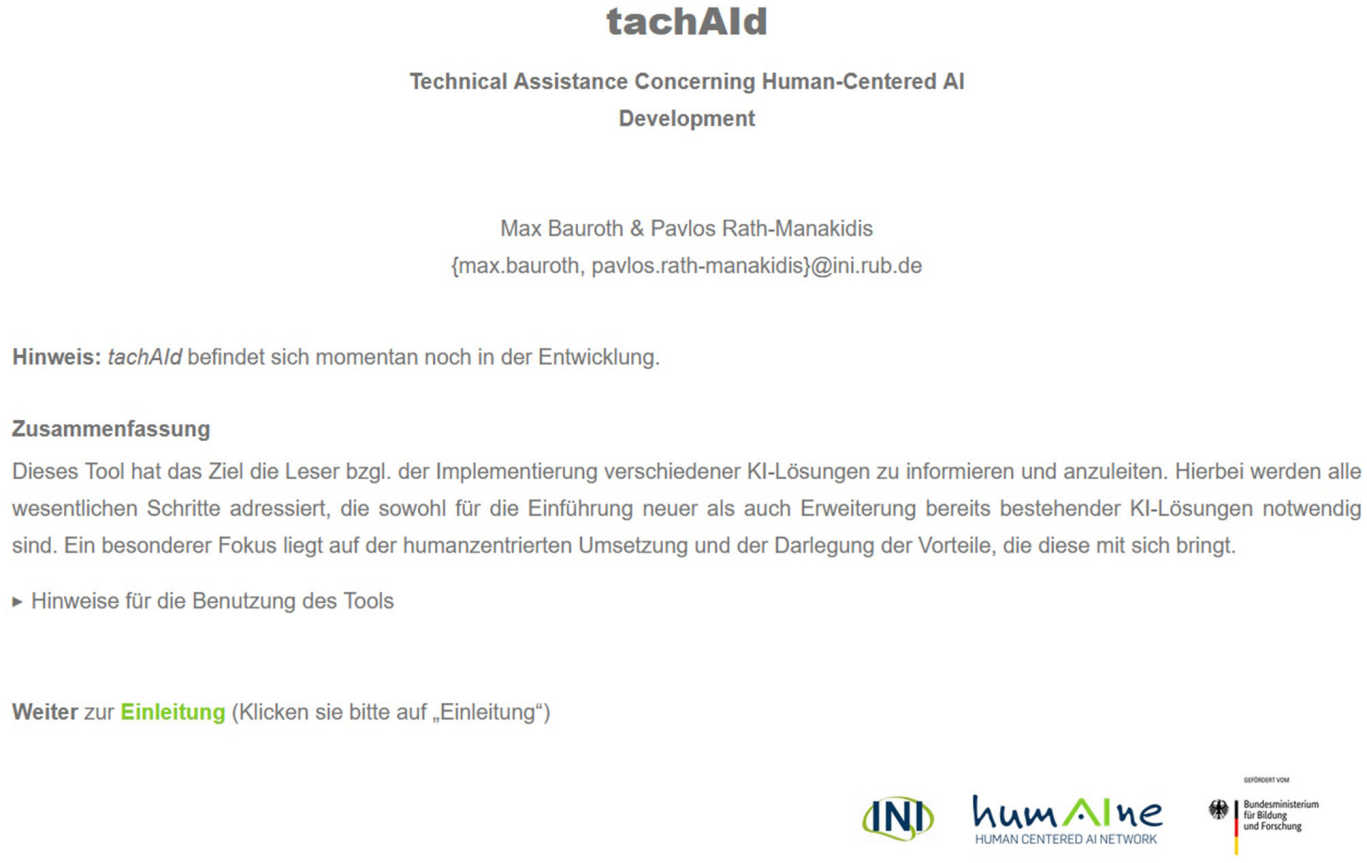
The first view of tachAId that is presented to the user, including a brief introduction to the tool. Optional instructions for using tachAId are available but hidden unless requested.

**Figure 3:**
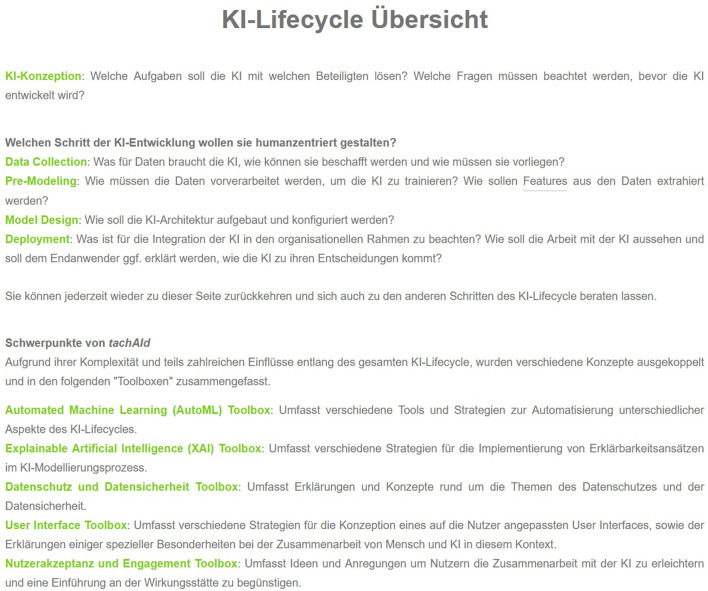
The view of tachAId that provides an overview of the AI lifecycle and the toolboxes included in tachAId. It serves as a hub for all sections of the tool and the user is directed back to this view after fully exploring a phase of the AI lifecycle.

Figure 2 shows a screenshot of the starting point of the tool and Figure 3 the overview of the AI lifecycle and the toolboxes, i.e., the contents of tachAId. Navigation through tachAId is done through user-driven exploration. The tool uses flow-based navigation, with click-based interactions and backward and forward buttons. The user is presented with a few short paragraphs of information at a time about the HCAI challenges faced in the current part of the AI lifecycle, and the primary means of navigation are highlighted passages or words that point to possible next steps or considerations. However, the tool is designed to allow for global hierarchical navigation so that the users can easily access specific sections or revisit earlier stages of the AI design process.

In the current iteration of the tool, a thorough session with tachAId can take up to two to three hours.

### 2.2 Validation framework

In this section, we present the framework that we developed in order to derive and evaluate the challenges and goals that ultimately shaped the contents of tachAId. In order to assess the quality of our tool and to determine (1) the thematic relevance of the specific challenges and goals presented in the context of human-centered AI within the work environment and (2) if the proposed measures to address these challenges and goals align with the ambitions and intentions formulated in the introduction, we derive and then propose an appropriate evaluation methodology. The final methodology takes the shape of a validation catalog for HCAI tools and will be presented later. It is built upon a framework that represents our understanding and definition of designing HCAI. This understanding is strongly motivated by the current literature on the topic. The basis for this framework is the work of Auernhammer (2020), Degen and Ntoa (2021), and Xu (2019). These authors subdivide the field of HCAI into three disciplines, i.e., areas of consideration and expertise, namely: ethics, technology, and essentially some form of human-AI interaction (HAII).

In this context, ethics is concerned with moral, societal, and legal norms and is the discipline through which relevant human needs enter into consideration. Technology refers to—often invisible—technical processes and performance measures and depends on the requirements derived from ethics and HAII, at least when executed from a design thinking perspective. Technology can inspire novel capabilities for those who work with it, but technical limitations may necessitate tradeoffs that need to be resolved by involving the other two sub-disciplines. HAII refers to the study, design, and implementation of interfaces and interactions between humans and artificial intelligence systems. It encompasses a wide range of topics and considerations aimed at facilitating seamless and effective ways for humans and AI technologies to communicate, collaborate, and cooperate.

Next, we attempted to determine the primary pillar of this framework—assuming one exists—i.e., the discipline that most shapes the pursuit of human-centeredness in HCAI and therefore requires more attention than the others. We approached this matter in a highly simplified and abstract manner. We intuitively assessed how much the human is involved in each subdiscipline, simply based on their general contents and goals.

To begin, technology, in its described form, completely ignores the human as a direct influence. The sole focus is on performance, quality measures, and the models used, leading to a complete neglect of people as a significant criterion.

In HAII, on the other side, the human is inevitably involved in some way. Depending on how much focus one wants and needs to give to this factor, its importance varies. However, if the goal is to be human-centered, it must be at least as important as the AI, if not more so.

Finally, there is ethics, which is exclusively human-centered and deals with the preconditions for and evaluation of human actions. By its very definition, its only focus is the human and therefore, from this abstract point of view, it is clear that ethics must be seen as the main motivator or driver for HCAI. In other words, it serves both as a guideline for the development of appropriate criteria that are crucial for the fulfillment of HCAI, and as a touchstone or measure respectively for assessing the relevance of challenges and goals derived from the other disciplines in the context of a human-centered development of AI.

This last fact is particularly important because the three subfields of this framework cannot be viewed as being isolated from one another. There are relationships between these issues that need to be considered.

For example, certain technical requirements that may initially appear to be unrelated to HCAI may prove to be relevant when evaluated from an ethical perspective, such as reproducibility of solutions. In this case, to provide the necessary assurance when AI interacts with human life in a potentially critical capacity, the behavior of the AI must be identifiable and predictable under repeated similar conditions. Then, of course, technology and HAII are inherently linked, whereby demands arising from the interplay between human and AI can have implications on the exact way how the technology has to be designed. Ethics and HAII are also related because the way the AI communicates with humans should, and sometimes must, be designed to meet ethical standards; for example, when physically or mentally impaired users interact with this technology.

Given that we defined ethics as driving force in HCAI, its content, articulated as guidelines and recommendations, shapes the very way in which the challenges and requirements arising from the other two disciplines are to be addressed. In particular, fundamental ethical principles are being used down the line to judge the specific challenges and goals stemming from each of those subfields, in order to evaluate their relevance and eligibility in a human-centered context. Furthermore, the fundamental ethical principles will be used to derive said challenges and goals, acting as the aforementioned guidelines and recommendations for how things should be considered in HCAI.

Following this logic, the next step is to define these ethical principles. Much work has already been done in this area. The work of Floridi et al. (2018) is noteworthy in this respect, as it compiles and compares several ethical principles from numerous publications and finally finds their counterpart in the four classical core principles of bioethics: beneficence, non-maleficence, respect for autonomy, and justice, which were first introduced in this concise form by the work of Beauchamp and Childress (2001).

Floridi et al. (2018) note that this area of applied ethics is particularly suited to dealing with new forms of technology that have some kind of impact on the animated environment, and thus can also be applied to AI.

According to Varkey (2020) *beneficence* is originally the principle that imposes moral rules on the active actor in the relationship in question to protect the rights of those affected, to avoid harm, to eliminate circumstances that could cause harm, and to provide special care and assistance to impaired individuals. In contrast to the principle of non-maleficence, the focus here is not simply on avoiding harm, but on actively acting for the benefit of those affected. In the literature, this principle is articulated in a variety of ways in the context of AI, but can generally be summarized as “AI should benefit the wellbeing of people and the planet, both now and in the future” (Floridi et al., 2018).

*Non-maleficence* is the obligation not to harm a person, either mentally or physically. In addition, the affected party should not be offended and his or her property should not be damaged (Varkey, 2020). In other words, AI systems should not harm humans or the environment in any way. Human dignity and the right to privacy are also affected by this principle. Specifically, non-maleficence also extends to harmful external influences, threatening the users via the AI (Beauchamp and Childress, 2001; EU, 2019).

According to the definition of autonomy by Kant (Guyer, 2003), *respect for autonomy* states that all persons have intrinsic and unconditional value and should therefore be able to make rational and moral decisions and be allowed to exercise their capacity for self-determination. Floridi et al. (2018) point out that this principle is more complex in the context of AI. According to this principle, the integrity of one's autonomy should be preserved, guarded, and respected. Only in certain and well-defined situations is it possible to overrule another person's autonomy. However, using AI involves humans willingly surrendering some of their autonomy to this technology, blurring the line of how exactly AI should respect the user's autonomy. The consensus in the literature they reviewed is that, at any given time, humans should be able to relinquish or regain exactly that part of their autonomy that they previously possessed or relinquished. In this sense, human autonomy should be promoted. The EU Ethics Guidelines for Trustworthy AI (EU, 2019) describe autonomy as ensuring human oversight over the work processes carried out by AI systems.

*Justice* is generally defined as fair and impartial (Varkey, 2020). In AI contexts, perspectives vary on justice, with some viewing AI as a remedy for past injustices, like discrimination. There's an understanding that AI's use should yield benefits shared equitably (Floridi et al., 2018). In the EU guidelines (EU, 2019), fairness supersedes justice, encompassing similar principles. Specifically, it argues that the development, deployment and use of AI systems should be fair. Benefits and costs should be shared equally, and there should be no unfair bias, discrimination or stigmatization. In particular, reference is made to AI practitioners who should carefully balance competing interests and goals. It is also noted that it should remain possible to challenge decisions made by AI systems and those who operate them and seek effective redress.

Apart from these four widely accepted core principles, Floridi et al. (2018) argue that they are not sufficient to cover the whole range of ethical issues related to AI and additionally proposed a fifth principle: *explicability*.

According to them, explicability encompasses two fundamental dimensions: intelligibility, delving into the AI's inner workings, and accountability, probing the question of responsibility for its performance. This principle completes the four core principles from bioethics by complementing each of them by answering the key questions of function and accountability. The EU Ethics Guidelines for Trustworthy AI (EU, 2019) also incorporate this principle, arguing that explicability is crucial to building and maintaining user trust in AI systems. Transparency and the ability to understand not only the system, but especially its decisions are essential to this end. Additional measures to ensure control and oversight are also relevant, especially if it is not possible to explain all of the AI's behavior, such as traceability, auditability, and transparent communication of the system's limitations and capabilities. They also emphasize that explicability is highly dependent on the context and consequences of the AI deployment, and thus the extent to which it is implemented must be decided on a case-by-case basis. Interestingly, this principle can also be motivated in its basic ideas by the fusion of two other bioethical principles, “informed consent” and “truth-telling” (Varkey, 2020). The former implies that the user of the system in question needs full knowledge or disclosure of the system and its effects. Furthermore, the user must be able to understand the explanation. This means that the way the information is presented must be adapted to the user's needs and abilities. Finally, “truth-telling” refers to the principle that in order to build trust in the AI system, its user must be assured not only that he is receiving all the information he needs, but also that he can fully rely on its validity.

Naturally, there are and will be conflicts between these core principles. For instance between the principle of beneficence and the principle of autonomy. As exemplified in the EU Ethics Guidelines for Trustworthy AI (EU, 2019), by an AI providing predictive information to prevent crime, using means which infringe personal autonomy like surveillance activities. As stated there and also in Varkey (2020), there is no silver bullet to resolve every possible conflict between these principles. A lot of the time they have to be solved on a per-case basis, using sound judgment and carefully weighing the benefits and drawbacks of every decision.

Using the three subfields ethics, technology and HAII, as well as the ethical core principles to judge and create challenges and goals in these fields, our framework is nearly complete. But, as mentioned in the introduction, tachAId has the distinct ambition to facilitate the adoption of AI in companies, ensuring that the humans are being sufficiently considered as early as the design process. Therefore, special attention must be given to this topic already on the level of the framework.

As of now, most literature primarily concerns itself with HCAI in a broader context, paying little attention, at best, to the labor environment in particular. In order to focus more on the latter, we extended our framework based on the work by Wilkens et al. (2021). They differentiate the valid ways to approach and understand AI in the labor context into a taxonomy of perspectives specific for AI to be used in the workplace. These perspectives can be used much like the ethical principles to both judge existing challenges and goals, but this time specifically regarding their suitability for AI in a company domain, as well as identify new challenges and goals, designed to consider the working environment.

**Figure 4:**
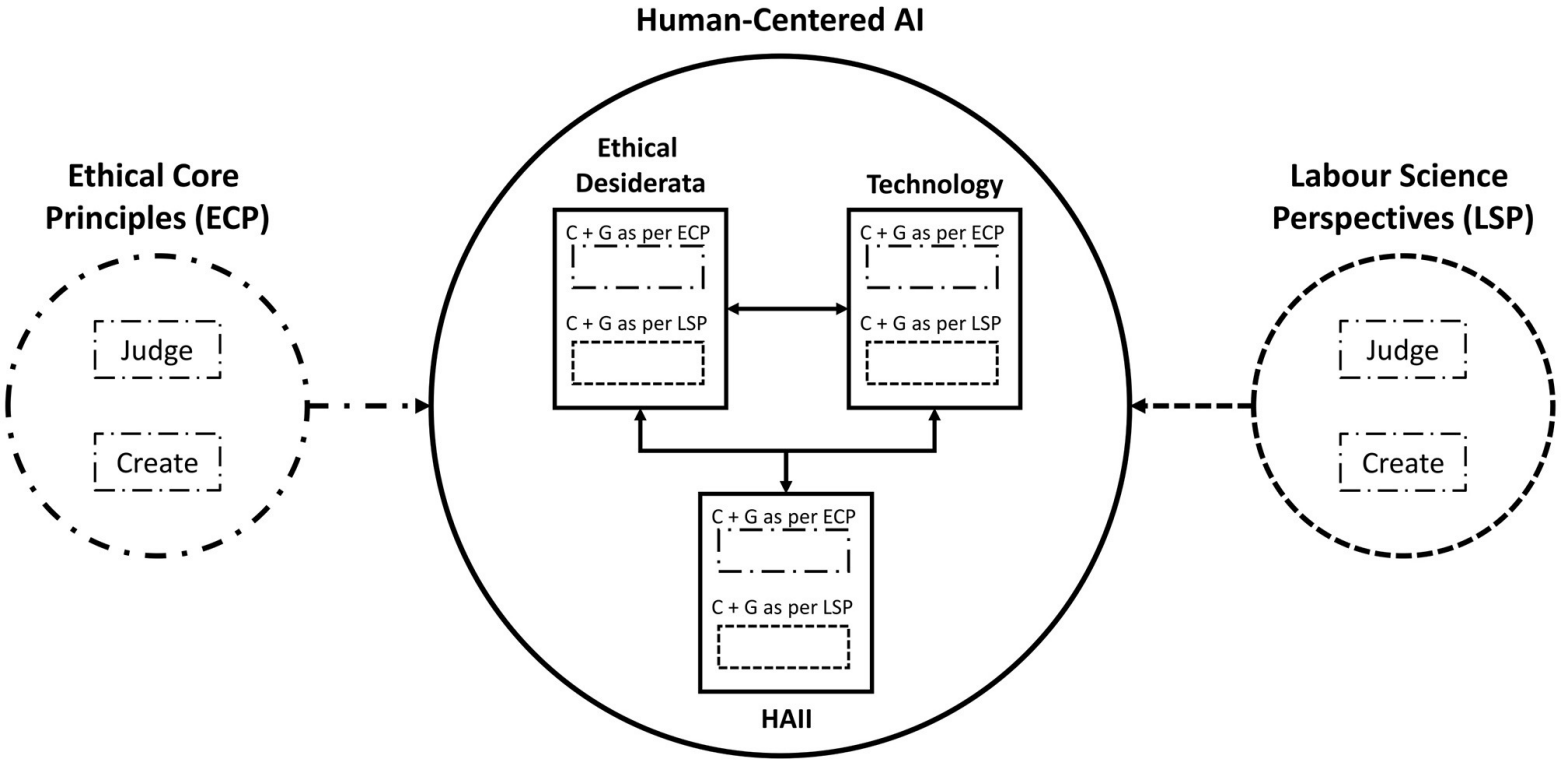
Depiction of the Framework. The three fields Ethical Desiderata, Technology and Human-AI Interaction (HAII) form the basis of Human-Centered AI and stand in relationship to each other. C, Challenges that arise in the respective field; G, Goals as in the specific goals that correspond to the respective challenges; ECP, Ethical Core Principles according to Floridi et al. (2018); LSP, Labor Sciences Perspectives according to Wilkens et al. (2021). “Judge” means that the challenges and goals from the individual fields are being judged both by the ECP and the LSP, respectively, in order to analyze whether they're appropriate for the respective field under a human-centered approach to AI and a work context specific approach. “Create” means that separate challenges and goals are being created under the ECP and LSP respectively. The different border styles highlight the relationship between ECP, LSP and the challenges and goals created within the three fields.

Summarizing the concepts outlined above, we derived our framework, depicted in its entirety in Figure 4. Shown are the three main fields of HCAI: Ethics, Technology, and HAII. They all influence each other, depicted by the double headed arrows. The five core ethical principles *beneficence, non-maleficence, respect for autonomy, justice*, and *explicability* are being used to create and judge challenges and goals arising in each of the respective fields. The relationship between those challenges and goals and the ethical principles is depicted by their matching borders. Finally, the five understandings (abbreviated as “U” in the following) from Wilkens et al. (2021): (U1) *deficit-oriented*, (U2) *data reliability-oriented*, (U3) *protection-oriented*, (U4) *potential-oriented*, and (U5) *political-oriented* are being used in much the same way, but under the scope of a workplace perspective. Their affiliation is also depicted by their matching borders.

### 2.3 Validation catalog

With the framework as a foundation, the validation catalog can be designed. Specifically, the catalog contains all the particular challenges and goals that need to be met by guidelines, tools, or other aids that aim to advise their users on how to design a human-centered AI; in our case, with a special consideration of a work environment.

Already many guidelines, rules and regulations have been proposed to help design HCAI friendly technologies, outlining the necessary steps in great detail. For example, important preliminary works by Amershi et al. (2019), Microsoft (2022), the EU (2019), and IBM (2022a) provide a good starting point for further analysis. However, none of these four guides have a strong focus on the use of AI at work. Even more, the majority of proposed works in this field either neglect this point of view entirely, or treat it very shallowly.

These aforementioned works serve as the primary references for the later concrete formulation of the challenges and goals in our developed tool. In particular, the framework of the EU Ethics Guidelines for Trustworthy AI (EU, 2019) largely overlaps with ours, so much so, that our framework can be seen as an extension of theirs.

Because of that and after reviewing their specific challenges and goals, we decided to primarily use them, adjusting and extending them to our needs and our slightly modified approach. In particular, the latter part – shifting the focus from general HCAI to HCAI in the work context and incorporating the work of Wilkens et al. (2021) – led us to not only devise a completely new challenge, but also to generally overhaul the set of proposed goals.

In detail, the EU Ethics Guidelines for Trustworthy AI (EU, 2019) identified seven challenges (hereafter abbreviated as “C”), i.e. requirements, for trustworthy AI: (C1) Privacy and data governance, (C2) Societal and environmental well-being, (C3) Diversity, non-discrimination, and fairness, (C4) Accountability, (C5) Technical robustness and safety, (C6) Human agency and oversight, and (C7) Transparency. Additionally, due to our modified work-centric approach, we added an eighth challenge (C8) User Adoption and engagement.

The individual challenges and the associated goals (hereafter abbreviated as “G” followed by the corresponding number of the challenge) are described in more detail below. Supplementary Table 1 presents the assignment of ethical principles and workspace-related understandings. Since in most cases it is reasonable to argue that each goal is motivated to some extent by all ethical principles and labor science understandings, we only note the ones with the best fit with the respective goal.

#### 2.3.1 (C1) Privacy and data governance

The challenge of privacy and data governance in human-centered AI at work aligns with the ethical principles non-maleficence, autonomy, justice, and accountability. These principles guide goal creation and ethical evaluation. Three understandings—data reliability, potential, and political—contribute to the goals by promoting data reliability, ensuring access and utility, and ethically controlling data and technology.

G1.1) Respect for privacy and data protection. This AI design goal involves responsible data handling to protect personal data throughout the AI lifecycle, preventing misuse and discriminatory actions. Emphasizing ethical data management builds trust, ensures user privacy, and avoids harmful consequences, essential in sensitive contexts.

G1.2) Quality and integrity of data. Ensuring AI systems train on diverse, reliable, and representative data is crucial. Data quality profoundly impacts AI performance, so errors like imbalances and inaccuracies must be removed before training. Data integrity is critical to prevent malicious inputs altering AI behavior. Thorough testing and documentation are essential at every stage.

G1.3) Access to data. This design goal establishes robust data management practices and transparent data audit trails. It involves creating data access protocols that delineate authorized personnel and their permissions.

#### 2.3.2 (C2) Environmental and societal wellbeing

Considering the challenge of environmental and societal wellbeing in the workplace, AI innovation must prioritize sustainability, environmental friendliness, positive working conditions, and societal impact. The ethical principles of beneficence, non-maleficence, respect for autonomy, and justice are hereby as important as the understandings U1, U3, U4, and U5.

G2.1) Sustainable and environmentally friendly AI. AI should minimize energy consumption, reduce the carbon footprint and appeal to socially responsible stakeholders. This involves using energy-efficient hardware, optimizing algorithms, and encouraging developers to create efficient solutions, leading to cost savings.

G2.2) Positive social impact on working conditions. Designing workplace AI for a positive social impact on the employees is crucial, fostering a healthier environment and improving their wellbeing. This includes aligning tasks with individual strengths, promoting a sense of accomplishment, providing varied and suitable assignments, and preserving job identity. AI can support a workers' continuous growth by adapting tasks to their evolving abilities and needs. By embracing these principles, AI becomes a catalyst for positive change, improving job satisfaction, overall productivity, and the overall quality of work life.

G2.3) Positive societal impact. Creating AI with a positive wider societal impact involves aligning technology with ethical and societal values, reflecting the needs of stakeholders. This AI fosters collaboration, equality, and transparency, enhancing organizational culture and promoting a sense of meaning.

#### 2.3.3 (C3) Diversity, non-discrimination, and fairness

This challenge aims to avoid bias, promote accessibility, and ensure representation and participation, guided by the principles of non-maleficence, respect for autonomy, and justice. It draws on specific understandings in human-AI teaming—data reliability, protection, and political—, seeking an inclusive, unbiased, and equitable work environment.

G3.1) Unfair bias avoidance. Critical for equality, avoiding bias ensures equitable decision-making and fosters inclusion. By preventing biased outcomes from factors such as faulty data or algorithms, AI contributes to organizational performance and employee wellbeing, valuing each individual equally.

G3.2) Accessibility and universal design. Creating universally accessible AI is essential for inclusivity and equity, allowing all individuals to benefit equally. Adhering to universal design principles ensures usability for everyone, enhancing participation, productivity, and high morale among the employees. Accessibility meets ethical and legal requirements, contributing to a diverse and harmonious workplace. Accessibility pertains to both the interface to the AI (e.g., intuitive interface, clear presentation of model outputs, and understandable explanations of model behavior), as well as to the AI's own capabilities.

G3.3) Stakeholder participation. Crucial for holistic success, involving end-users, developers, management, and experts ensures diverse perspectives, fosters innovation, and proactively addresses potential issues. This participatory approach enhances user satisfaction, minimizes resistance, and aligns AI with organizational goals, contributing to successful integration into the workplace ecosystem.

#### 2.3.4 (C4) Accountability

Addressing accountability in Human-Centered AI, particularly in the workplace, focuses on creating transparent and scrutinizable AI systems that minimize harm, address ethical complexities, and offer user recourse. It emphasizes the need to protect human wellbeing and establish dependable conditions for potential unleashed through good governance, reliability, and trust. Key understandings in a workplace context include protection-oriented (human wellbeing), potential-oriented (benefits of AI-human collaboration), and political-oriented (power dynamics) understandings. From an ethical point of view, explicability, non-maleficence, and justice are the most important factors.

G4.1) Auditability. Critical for transparency, accountability, and compliance, an auditable AI allows tracking and verifying decision-making processes for ethical outcomes. Documenting algorithms, data sources, and decision paths builds trust with users, stakeholders, and regulators, promoting ethical use and a work environment based on accountability.

G4.2) Minimizing and reporting negative feedback. Creating an AI for the workplace that facilitates reporting and responds to negative user feedback is crucial for continuous improvement. Addressing concerns in a timely manner enhances user satisfaction, loyalty, and fosters a culture of open communication, problem-solving, and refinement for a more user-centric work environment.

G4.3) Addressing trade-offs related to AI. Navigating conflicts between AI requirements and adherence to core ethical principles ensures balanced decisions and ethical outcomes. Managing trade-offs between efficiency, fairness, and moral values safeguards against biased or harmful decisions, reinforcing accountability and fostering a workplace that values integrity and ethical responsibility.

G4.4) Ability to redress. Focusing on recourse and complaint mechanisms, this goal ensures affected parties can seek redress for transparency and accountability. Providing avenues for redress builds trust, upholds fairness, protects individual rights, and promotes a responsible work environment that values correcting and learning from mistakes, fostering a culture of fairness and equity.

#### 2.3.5 (C5) Technical robustness, safety, and performance

This challenge is a primary concern in Human-Centered AI, addressing goals to ensure AI system resilience, manage contingencies, enhance accuracy and performance, and ensure reliability. Shaped by the maxims of beneficence, non-maleficence, justice, and explicability, as well as the understandings U2, U4, and U5, these goals aim to safeguard the AI system against threats and foster trust among stakeholders.

G5.1) Resilience to attack and security. A fundamental design consideration for workplace AI, this goal involves building defenses against intentional and unintentional threats. Securing the AI system protects operations, data integrity, and prevents unauthorized access. Prioritizing this goal ensures productivity, protects sensitive information, and maintains trust in the workplace.

G5.2) Contingency management and fallback measures. Critical for business continuity, contingency management and fallback measures reduce disruptions due to AI malfunction. Anticipating and remedying failure cases, these measures enable the AI system to return to a safe state and mitigate errors, supporting uninterrupted workflow and a safe, resilient, and efficient work environment.

G5.3) High accuracy and performance. Assessing performance in human-AI collaboration, this goal emphasizes reliable and sustained performance considering complementarity and potential friction. Leveraging AI precision and human creativity enhances productivity, reduces errors, and contributes to workplace success.

G5.4) Reliability and reproducibility. Creating reliable workplace AI with reproducible behaviors is critical for consistent performance and problem-solving. Prioritizing reliability and reproducibility increases efficiency, minimizes disruption, and fosters a work environment where results can be replicated, reviewed, learned from, and trusted.

#### 2.3.6 (C6) Human agency and oversight

Establishing human agency and oversight is a crucial challenge in human-centered AI in the workplace, balancing individual empowerment and responsible AI integration. The relevant goals G6.1 to G6.3 establish the primacy of human reason and human goals over AI. These goals resonate hereby with the principles of non-maleficence and respect for autonomy, and the workplace related perspectives—protection, potential, and political—confirm their relevance.

G6.1) Ensuring fundamental rights. Ensuring fundamental rights, such as privacy and non-discrimination, affirms individual agency and dignity. This goal ensures fair treatment, legal compliance, trust, and reliability while avoiding reputational risks for the company.

G6.2) Human agency. Facilitating and safeguarding human agency is crucial for ethical AI integration, ensuring AI supports human decision-making without replacing or controlling it. Prioritizing human agency minimizes the risk of biased or harmful autonomous AI decisions, promoting accountability, trust, and transparency in the human-AI relationship.

G6.3) Human oversight. Designing AI with human oversight is essential for accountability, ethical decision-making, and maintaining control. This goal allows human intervention and correction, preventing unintended consequences, errors, or biases. Facilitating human oversight ensures ethical boundaries, transparency, and a collaborative human-AI partnership that promotes responsible workplace technology use.

#### 2.3.7 (C7) Transparency

In the realm of HCAI in the workplace, transparency is a crucial consideration with goals driven by key ethical principles—beneficence, respect for autonomy, and explicability. Additionally, with the understandings U2, U3, U4, and U5, these goals aim for a transparent landscape that facilitates harmonious interaction between humans and AI, and do so by seeking to foster a deeper understanding of and trust in it.

G7.1) Traceability. Tracking model decisions and data points through AI and data pipelines, including collection, annotation, pre-processing, as well as model design options is critical for transparency, accountability, and error mitigation. Traceability enables understanding AI decisions, identifies biases or errors, fosters trust, supports compliance, and ensures continuous improvement based on data-driven insights.

G7.2) Explainability. Focused on building trust and user acceptance, explainability ensures that AI decisions are understandable and justifiable. This goal enhances predictability, identifies biases and errors for timely corrections, and fosters a collaborative partnership between humans and AI, promoting productivity and informed decision-making.

G7.3) Foster AI awareness, communicate limitations, provide decision feedback. Essential for trust and responsible use, transparency in the workplace involves revealing AI presence, nature, and limitations. This empowers users, sets realistic expectations, promotes accountability, and informs decision-making. User feedback increases transparency, builds trust, and enables effective collaboration, creating a balanced human-AI partnership.

G7.4) Intuitive user experience and effective user interface. A well-designed interface enables seamless interaction, increasing user satisfaction and accessibility for non-technical users. Intuitive design reduces training time and errors, improving productivity, user sentiment, and encouraging wider adoption. Effective UI design ensures easy navigation and utilization of AI capabilities, optimizing workflow efficiency and supporting a positive work environment.

#### 2.3.8 (C8) User adoption and engagement

Adapting the EU Ethics Guidelines for trustworthy AI (EU, 2019) to the workplace, this challenge focuses on considering user motivation, onboarding, and retention, rooted in beneficence, respect for autonomy, and explicability. Drawing from data reliability-oriented and potential-oriented perspectives, it aims to improve AI reliability and foster collaborative human-AI engagement for an inclusive and harmonious work ecosystem.

G8.1) Education and onboarding. Introducing AI to the workplace involves providing user training and orientation to maximize potential and minimize resistance. Training builds skills and confidence, while orientation reduces resistance, communicates benefits, and ensures a smooth transition. This approach accelerates AI adoption, fostering a positive user experience and optimizing its contribution to productivity and innovation.

G8.2) User engagement. Ensuring long-term user engagement with AI through mechanisms, elements, and incentives drives continuous interaction and learning. Regular interaction deepens familiarity and trust in AI capabilities. Fostering a positive attitude and sense of ownership increases user adoption and leads to continued, enthusiastic use. Sustained engagement creates a positive feedback loop, boosting user satisfaction and AI utility for both users and the organization.

An overview of the collected challenges and goals, with brief definitions, is presented in Supplementary Table 2.

## 3 Results

In this section, we show the extent to which tachAId meets the validation criteria formulated in Section 2.3. For the sake of comparison, we also validate the EU Ethics Guidelines for Trustworthy AI (EU, 2019) in the same way.

The results are shown in Table 1. We evaluate the degree to which each tool fulfills each goal by how concrete the advice the tool provides is. We have chosen a symbol-based coding to clarify whether a goal was fully met (✓), half met (✓/2), or not met at all (x). Here, we define “not at all fulfilled” as the case that the problem is not mentioned at all in the tool; “half fulfilled” as the case that at least the significance of the problem is emphasized in the tool; and “completely fulfilled” as the case that both the significance of the problem is discussed in the tool and at least one concrete solution proposal is presented.

**Table 1:** Comparison of tachAId and the EU Ethics guidelines for trustworthy AI (EU, 2019) with the goals of the validation catalog defined in Section 2.3.

**Challenges**	**Goals**					
	**EU guidelines**			**tachAId**		
	**x**	✓**/2**	✓	**x**	✓**/2**	✓
(C1) Privacy and data governance	-	1.1, 1.2, 1.3	-	-	-	1.1, 1.2, 1.3
(C2) Environmental and societal wellbeing	-	2.1, 2.2, 2.3	-	2.1, 2.3	2.2	-
(C3) Diversity, non-discrimination, and fairness	-	3.1, 3.2, 3.3	-	-	3.2	3.1, 3.3
(C4) Accountability	-	4.1, 4.2, 4.3, 4.4	-	4.4	4.2	4.1, 4.3
(C5) Technical robustness, safety, and performance	-	5.1, 5.2, 5.3, 5.4	-	5.2	5.4	5.1, 5.3
(C6) Human agency and oversight	-	6.1, 6.2, 6.3	-	6.1	-	6.2, 6.3
(C7) Transparency	-	7.1, 7.2, 7.3, 7.4	-	-	7.3, 7.4	7.1, 7.2
(C8) User adoption and engagement	8.2, 8.3	-	-	-	8.2, 8.3	-

On the left side, the challenges relevant to the design of a human-centered AI are listed. On the right side, the goals defined under the challenges in section 2.3 are abbreviated by their respective number and assigned to the symbols (x, ✓/2, ✓) under the respective tool/guideline where they fit best. The symbols indicate whether a goal is not fulfilled at all (x), partially fulfilled (✓/2) or fully fulfilled (✓).

## 4 Discussion

As AI becomes more and more integrated into our everyday lives, the need for regulation and governance in the sense of a human-centered approach is becoming ever stronger. Against this background, some works (Amershi et al., 2019; EU, 2019; Degen and Ntoa, 2021; IBM, 2022a; Microsoft, 2022) have already tried to investigate and establish clear rules and guidelines for the design of HCAI solutions, which addresses issues as early as the first stages of conceptualization and thus try to help the user consider the human in their solution and highlight potential pitfalls during the design of the AI. However, these guidelines often disregard the work environment, the importance of which we emphasized in the introduction of this paper and is based on the fact that we spend a large part of our lives at work (Di Battista et al., 2023; Hatzius et al., 2023) and that human-friendly and human-centered work environments require special consideration (Wilkens et al., 2021; Berretta et al., 2023).

### 4.1 Organizational prerequisites for using tachAId

In the design of tachAId we assume that certain steps toward the introduction of AI have been taken on an organizational and technical level, as a prerequisite for using tachAId to reliably introduce AI into work processes. We discuss these steps in this section. Nonetheless, tachAId can be used to gain insight into the challenges of integrating AI into work processes even if these conditions are not met.

First, a solid foundation in digitization and data management is essential, including digital infrastructure and competencies and data repositories with relevant, qualitative, complete, and diverse data. Tools from labor psychology such as the Job Perception Inventory (JOPI) (Berretta et al., 2023) help to understand employees' perceptions and needs during the AI implementation process and the possible change in their attitude toward AI in the course of AI introduction.

An AI-Maturity analysis is crucial for evaluating the organization's readiness and potential (Bülow et al., 2022) to embrace AI and entails assessing change management strategies. It is also beneficial to involve employee representation or participation to foster inclusive AI development.

Furthermore, a clear vision for introducing AI into value-added processes is necessary. This includes defining the desired AI function (i.e., outputs) and considering how to measure the performance of the AI-enhanced work process. Adequate preparation of training data and validation of model behavior will require dedicated resources and skilled personnel.

When incorporating AI into work processes, special consideration must be given to human-AI interaction. Determining the order in which humans and AI systems act, the depth of their interaction, and the mode of collaboration between the two is key to ensuring productive and harmonious coexistence. Interaction design is an opportunity to convert informal knowledge into process specifications or best practices and support employee engagement, creativity, and agency. Mock-ups and Wizard of Oz experiments that prototype human-AI workplaces can provide insights into the opportunities and pitfalls of human-AI arrangements, and clarify which tasks should or should not be automated.

### 4.2 Discussion of the form and interface of tachAId

As one of the main contents of this work, we have presented tachAId, an interactive, HTML-based and browser-compatible tool that aims to provide developers, users and in general stakeholders in companies with a guide so that AI can be implemented in a social and ethical way that is beneficial to everyone involved. The structure of tachAId is based on the CRISP-DM model (Shearer, 2000) and leads sequentially from data collection to deployment. General concepts, which are not clearly assigned to one of the main phases of AI development, or specialized content, are offered in so-called toolboxes. The interactivity of tachAId encourages the user to think more deeply about the human-AI interaction requirements for their solution, and to obtain and consume more relevant information than would be the case with simple linear text. Due to the way tachAId is designed, the content is highly structured, protecting the user from being overloaded with information, instead presenting them with only what they need at any given moment. On the downside, tachAId in its current form lacks a glossary, which will be added in the future, so it is not possible to search for specific content. This is a disadvantage compared to simple linear documents. Furthermore, as the tool's content becomes available only as the user interacts with it, users may not be able to access certain information relevant to them if they stop using the tool prematurely. A general high-level overview of the content of tachAId in a suitable place can address this problem. Further improvements can and will be made to the tool's navigation. It currently contains only rudimentary elements for navigating between pieces of information and re-accessing previously accessed information. Finally, the way content is presented with respect to the technical expertise of its users should and will be modified in the future by hiding advanced information for users who do not want or understand more in-depth explanations of, for example, details about algorithms.

We observe two general disadvantages of the interactive format of tachAId. First, updating and expanding the tool requires not only researching and writing text, but also updating the website design and the available interactions to accommodate the nuances of different possible use cases. Second, a certain level of familiarity with digital media and an interest in working with interactive media form a barrier to using tachAId. This is especially true for those who are not familiar with human-computer interaction.

### 4.3 Discussion of the HCAI framework

The HCAI framework on the basis of which tachAId has been designed, is largely based on the work of Auernhammer (2020), Degen and Ntoa (2021), and Xu (2019). All three understand HCAI as an interplay of three essential components: ethics (moral, social, and legal norms), technology (technical processes and performance), and HAII (human interaction with AI). Next, we tried to identify the primary driver, i.e., the primary component in this interplay of factors, and to find the aspect that is most important—and therefore most worthy of attention—in shaping the human-centeredness claim in HCAI. To this end, we assessed in a very simple way how much the human is involved in each of the sub-disciplines, simply based on their general content and goals. Ethics was recognized as the most relevant component, in that it defines ethical principles as a driving factor that describes both critical guidelines that are essential for the full implementation of HCAI, as well as, a means to validate the challenges and objectives of the other two aspects of HCAI in terms of their relevance in this framework. The final five fundamental ethical principles were taken from the work of Floridi et al. (2018), which in turn are based on the prevailing principles of bioethics and extend them to include the point of explicability. This approach is largely identical to that of the EU Ethics Guidelines for Trustworthy AI (EU, 2019). In their work, the authors also use basic ethical principles to determine specific challenges and goals, which they ultimately use to define trustworthy AI. However, they only used four principles, excluding beneficence, although implicitly using it in the definition of their challenges and goals. We felt the need to explicitly include this principle, since although it is similar to non-maleficence, they are not identical in their effects.

Unlike (EU, 2019), we embed the subsequently defined challenges and goals in the academic HCAI framework we use. This is advantageous because it helps structure the problem of identifying and assessing challenges and goals, while being aware of their origin, and subsequently to work them out more clearly. Furthermore, we explicitly define the ethical principles as a criterion all possible challenges and goals must meet, including those from the areas of technology and HAII, in order to be recognized as human-centered and relevant for an HCAI in this sense. The results of this validation can be found in Supplementary Table 1.

Finally, given the scope of this paper and the fact that AI will change the business and work landscape in particular, we looked for ways to include workplace-specific perspectives at the framework level. In line with the criticism of the EU guidelines by Hickman and Petrin (2021)—who argue that the guidelines often lack detail or simply neglect certain topics from a company law perspective, and therefore fail to immediately aid in HCAI design—we draw on the work of Wilkens et al. (2021), who have explicitly placed this focus in their work and developed five understandings (deficit-oriented, data reliability-oriented, protection-oriented, potential-oriented, and political-oriented) that shed more light on this context. To verify the general applicability of this work-centered approach to our validation goals, we mapped the goals to matching understandings. The results for the most relevant matches can be found in Supplementary Table 1. This was possible for most goals, but for some it was difficult to connect them to any labor science understanding. One reason for this is that in the political-oriented understanding only the relationship between humans and AI is examined. However, against the background of a holistic company-centered approach, the relationship between the various stakeholders that interact with the AI is also important. The topic of trust is also limited to the data involved in AI and not expanded to the relationship between humans and AI. In general, however, this comparison confirmed the applicability of the understandings to our framework and thus allowed it to be expanded by a labor science perspective. The understandings, just like the ethical principles, were then defined as a force for elaborating and evaluating the challenges and goals stemming from the three main aspects of ethics, technology and HAII from a human factors perspective. The end result is a framework that is determined by two driving forces, the ethical principles and the labor science understandings.

### 4.4 Discussion of the validation methodology

Our validation catalog shares most challenges and goals with the EU Ethics Guidelines for Trustworthy AI (EU, 2019). We initially considered further literature (Baier et al., 2019; Cherrington et al., 2020; Margetis et al., 2021) as a reference for the evaluation of tachAId, but the EU guidelines proved to be the most comprehensive general reference. We saw fit to extend the EU guidelines by adding challenge (C8) user adoption and engagement and goals (G8.1) education and onboarding and (G8.2) user engagement. We have identified the need to consider the motivation and qualification of the user, because in the corporate context there is often no minimum level of knowledge or skill, either with software tools, let alone AI, that can be relied upon (Chaudhry and Kazim, 2022). Additionally, intensive and prolonged use of AI, especially in the context of monotonous tasks, requires a strategy for maintaining motivation in the face of fatigue. In establishing a concrete link to the world of work, we have reviewed each goal from this perspective and, where necessary, reformulated them accordingly. This is particular true of goal (G2.2) positive social impact on working conditions, which originally considered the social impact in a broad sense, and now addresses the specific challenges of a work environment, such as promoting job identity and motivation, and preventing fatigue and de-skilling.

We have examined tachAId in its current state with regard to the challenges and goals defined in Section 2.3. A three-tier grading is used to intuitively assess the extent to which they are implemented. We differentiate between the cases “not met at all”, “half met” and “fully met”. The degree of implementation depends on whether the respective goal is addressed comprehensively in the tool at all (if not, it is considered not fulfilled), and if so, whether at least one concrete solution is proposed for its fulfillment. If a solution is offered, then it is considered fully fulfilled, otherwise it is not. This is only a rudimentary analysis at this point, and a more detailed evaluation can and should be provided in the future if it proves useful. For example, the current definition of a fully met goal does not place any requirements on the quality of the solution provided. In addition, further nuances could be defined regarding the partial fulfillment of the goals.

### 4.5 Discussion of the results

Table 1 summarizes the extent to which tachAId achieves the goals from the validation catalog. The aim of tachAId is to bridge the gap between general principles for HCAI and specialized technical advice. To evaluate how well this is achieved for each of the goals identified for HCAI design, we assess whether each goal is introduced, motivated, and operationalized by providing advice or pointing to existing tools or methodologies.

We proceed to discuss how tachAId realizes some of these goals. G1.1 and G5.1 are dealt with concisely in the data protection and data security toolbox. The meanings of G1.2 and 1.3 and their specific implementation are clarified in the Data Collection section of tachAId. In the same section, the possible biases in the data and the employees working with the systems are also broken down and measures to avoid or reduce them are explained (G3.1). How exactly good stakeholder participation should look like (G3.3) and which strategies can be implemented to promote it are discussed in the section titled “conceptual questions and notes”. The goals of auditability and traceability (G4.1 and G7.1) are embedded in the tool by repeatedly reminding users to protocol the implemented procedures and the rationale that led to each technical decision. G4.3 is addressed by pointing out the advantages and disadvantages of each of the proposed procedures when a trade-off between human needs and values and AI requirements arises. To ensure a high level of accuracy and performance of the final AI system (G5.3), procedures are mentioned at the appropriate points along the ML design process that reinforce this objective. The topic of explainability (G7.2) is covered succinctly in the XAI Toolbox. With respect to the goals of human agency and human oversight (G6.2 and G6.3), the model selection section discusses the general principles and strategies for addressing this during the AI design. All goals pertaining to user interface and user interface design, these are G3.2, G4.2, G7.3, and G7.4, are so far only “half fulfilled”. Their importance and significance in the context of HCAI is highlighted in the user interface toolbox, but no solutions have been offered to date. The same applies to objectives G8.1 and G8.2. Although their importance is highlighted in the toolbox on user acceptance and engagement, no solutions are offered to implement them. Goal G5.4 (reliability and reproducibility) is also only half fulfilled. More specifically, in the deployment part, the retraining of the AI is mentioned as a possible strategy to ensure the reliability of the AI over a longer period of time, but the tool does not yet explicitly address how the reproducibility of ML results can be ensured. Finally, G2.2 is half-fulfilled by virtue of the AutoML toolbox, because AutoML is a tool specifically to improve the working conditions of AI developers, but no measures are presented on how AI can be used to improve the working conditions of end users. Goals 2.1, 2.3, 4.4, 5.2 and 6.1 are not yet addressed in any way in tachAId.

We have also examined the EU guidelines (EU, 2019) using our validation catalog and quantified the degree to which the goals are fulfilled. We found that it “half fulfilled” all goals, except for 8.1 and 8.2 which we introduce in this paper. The EU guidelines do not fully fulfill any goal, because they do not discuss any concrete proposals for solutions, but rather attempt to sensitize the reader to the goals of HCAI and their implications to the greatest possible extent by raising relevant questions.

Neither tachAId nor the EU guidelines offer exhaustive support for HCAI. tachAId mediates between general principles and requirements on the political and organizational level and measures for their technical implementation, while the EU guidelines focus on detailing the facets of the various challenges and goals that comprise HCAI on the conceptual level. Therefore, it is not possible to say whether tachAId or the EU guidelines are better suited to support the development of human-centered AI in general. Furthermore, it is often impossible to provide the full spectrum of solutions for the various problems, because the field of (HC)AI is too large, changing rapidly, and the individual customer requirements for the final AI are too diverse. For this reason, the tool presented in this paper, tachAId, can currently best be understood as an extension to the EU guidelines that specifically elaborates on practical considerations and measures for introducing AI in the workplace in an ethical and human-centered manner.

## 5 Conclusion

In this paper, we have delved into the pressing challenges of human-centered AI development and documented the need for practical advice grounded in ethical principles and comprehensive guidance to design AI solutions that cater specifically to human needs in work environments for AI developers and company decision-makers involved in the conception of AI tools. To address this need, we have introduced tachAId—an innovative interactive tool that provides technical assistance for human-centered AI development and discussed its key characteristics: its form, layout, and contents. In tachAId, potential challenges arising from the intersection of humans and AI are organized by the phase in AI development that they pertain to and are mapped to relevant technical measures and tools in a clear and engaging manner. In this way tachAId empowers AI development that aligns with human-centered objectives.

We designed our tool based on an extended HCAI framework presented in this paper, which uses both core ethical principles as well as labor science perspectives to define its HCAI-related challenges and goals. These challenges and goals comprise a validation catalog, which we used to examine the potential of tachAId and compare it to the EU Ethics Guidelines for Trustworthy AI. We conclude that tachAId is a valuable tool that can help developers, users, and stakeholders with practical advice in the development process of their individual AI.

### 5.1 Future work

Going forward, we primarily want to make tachAId more comprehensive and improve its interactivity. To this end, we will follow the validation catalog and target the challenges and goals contained therein and validate tachAId with a wide and diverse set of AI stakeholders. The validation catalog also provides a perspective for looking at other existing tools for HCAI, in order to generate new insights into the space of advisory tools, policy documents, and guidelines on HCAI and how they address the gap between the policy level and the day-to-day work of developing and designing AI.

To enhance the user experience, we intend to introduce additional navigation elements to complement the current flow-based design. This may include a sidebar that provides an overview of the user's position within tachAId's contents, making it easier to identify and access relevant topics and be able to ascertain that one has seen all contents in the tool. We also intend to make tachAId more stateful by increasing its awareness of the context and the goals of the user's AI project. Users will be able to provide project details such as AI application type, target audience, development context (in-house, by a contractor), and sensitivity of the application. Based on this information tachAId will be able to offer better tailored advice and content. A possible further development is to use this information to make tachAId a full-fledged recommender system in the technical sense.

## Data availability statement

The original contributions presented in the study are included in the article/Supplementary material, further inquiries can be directed to the corresponding author/s.

## Author contributions

MB: Conceptualization, Investigation, Methodology, Software, Validation, Visualization, Writing—original draft, Writing—review & editing. PR-M: Conceptualization, Investigation, Software, Validation, Visualization, Writing—original draft, Writing—review & editing. VL: Conceptualization, Funding acquisition, Methodology, Writing—original draft. TG: Conceptualization, Supervision, Writing—review & editing. LW: Conceptualization, Supervision, Writing—review & editing.

## Footnotes

^1^The current version of the tool is available on the web page humaine.info/tachaid-tool.
