# Supplementary material for: tachAId—An interactive tool supporting the design of human-centered AI solutions
Source: Front Artif Intell. 2024 Mar 12;7:1354114. doi: 10.3389/frai.2024.1354114 (PMC10963619; doi:10.3389/frai.2024.1354114)
Supplement: Supplementary file 1 [file Table_1.docx]

Supplementary Table 1: This table describes the relationship between the goals in the validation catalog and the ethical core principles and labor science perspectives, i.e. understandings, on HCAI in the workplace. We report the core ethical principles and workplace-related understandings that most strongly inform and help articulate and refine each goal for HCAI in a work context. We thereby summarize that the ethical core principles and workplace-related understandings justify and motivate each of the goals. Legend: Ethical Core Principles: (1) Beneficence, (2) Non-Maleficence, (3) Respect for Autonomy, (4) Justice, (5) Explicability. Labor Science Perspectives: (1) Deficit-Oriented, (2) Data Reliability-Oriented, (3) Protection-Oriented, (4) Potential-Oriented, (5) Political-Oriented. Numbers in bold indicate a high fit with the goal, while numbers in normal print indicate a moderate fit.

| Goals | Ethical Core Principles | Labor Science Perspective |
| --- | --- | --- |
| (G1.1) Respect for privacy and data protection | **2**, 3 | **3** |
| (G1.2) Quality and integrity of data | **2**, 4 | **2** |
| (G1.3) Access to data | 4, **5** | 4, **5** |
| (G2.1) Sustainable and environmentally friendly AI | **1**, 2 | 5 |
| (G2.2) Positive social impact on working conditions | **1**, 3 | 1, **3** |
| (G2.3) Positive societal impact | **1**, 4 | **4**, 5 |
| (G3.1) Unfair bias avoidance | **2, 4** | **2** |
| (G3.2) Accessibility and universal design | 3, **4** | 3, **4** |
| (G3.3) Stakeholder participation | **3, 4** | **5** |
| (G4.1) Auditability | 4, **5** | 3 |
| (G4.2) Minimizing and reporting negative impact | **2**, 5 | **3** |
| (G4.3) Addressing trade-offs related to AI | **2, 5** | 3 |
| (G4.4) Ability to redress | **4** | **5** |
| (G5.1) Resilience to attack and security | **2** | **2**, 3 |
| (G5.2) Contingency management and fallback measures | **2**, 5 | **3** |
| (G5.3) High accuracy and performance | **1**, 4 | **4** |
| (G5.4) Reliability and reproducibility | **2, 5** | **2** |
| (G6.1) Ensuring fundamental rights | 2, **4** | **5** |
| (G6.2) Human agency | **1, 3** | **4**, 5 |
| (G6.3) Human oversight | 2, **3** | **3** |
| (G7.1) Traceability | **3, 5** | **2** |
| (G7.2) Explainability | **3, 5** | **2** |
| (G7.3) Foster AI awareness, communicate limitations, decision feedback | **3, 5** | **5** |
| (G7.4) Intuitive user experience and effective user interface design | 1, 3, 5 | 3, **4** |
| (G8.1) Education and onboarding | **3, 5** | **2** |
| (G8.2) User engagement | **1, 3** | **4** |
