# Supplementary material for: tachAId—An interactive tool supporting the design of human-centered AI solutions
Source: Front Artif Intell. 2024 Mar 12;7:1354114. doi: 10.3389/frai.2024.1354114 (PMC10963619; doi:10.3389/frai.2024.1354114)
Supplement: Supplementary file 2 [file Table_2.docx]

Supplementary Table 2: This table summarizes the challenges (C), with their respective goals (G), matched to the different disciplines from the presented framework (see figure 4). Short definitions of the goals are provided.

| Discipline in Framework | Challenges | Goals | Goal Definition |
| --- | --- | --- | --- |
| Ethics | (C1) Privacy and data governance | (G1.1) Respect for privacy and data protection | Guarantee that the AI system handles personal data in a manner that respects the individuals' privacy and complies with data protection regulations. Minimize the use of sensible data for training. |
|  |  | (G1.2) Quality and integrity of data | Guarantee that the AI system is trained on sufficient and reliable data in a manner such that it is free from biases and inaccuracies. |
|  |  | (G1.3) Access to data | Implement proper data management practices and enable data auditability. |
|  | (C2) Environmental and societal well-being | (G2.1) Sustainable and environmentally friendly AI | Minimize the environmental cost of AI training, deployment, and use. Design the AI to promote environmentally conscious decisions. |
|  |  | (G2.2) Positive social impact on working conditions | Assess and manage the effects of the AI system on working conditions, including its impact on job satisfaction, qualification, and job security, to ensure a positive and empowering work environment. |
|  |  | (G2.3) Positive societal impact | Strive to create an AI that contributes positively to society, promoting inclusivity and social progress while minimizing potential harmful consequences. |
|  | (C3) Diversity, non-discrimination, and fairness | (G3.1) Unfair bias avoidance | Ensure that the AI system is developed and validated such as to avoid any unfair bias or discrimination based on individual characteristics. |
|  |  | (G3.2) Accessibility and universal design | Design the AI for broad user inclusivity. Prioritize accessibility. Pursue universal design norms and standards to ensure equal participation and inclusion of people regardless of their cultural or physical and mental differences or impairments. |
|  |  | (G3.3) Stakeholder participation | Actively involve all stakeholders, including end users, employees, and affected communities, in the design and deployment of the AI to integrate their needs and preferences into its design. |
|  | (C4) Accountability | (G4.1) Auditability | Ensure logging and the ability to inspect and verify the AI system's decision-making process and its data against current regulations. |
|  |  | (G4.2) Minimizing and reporting negative impact | Analyze and implement strategies to reduce harmful consequences resulting from the AI system and facilitate and provide comprehensive reports on such effects and incidents. |
|  |  | (G4.3) Addressing trade-offs related to AI | Address potential conflicts among the requirements of the AI system, identifying and evaluating trade-offs with respect to core ethical principles and performance measures. Decisions must be justified, documented, and continually reviewed for appropriateness and accountability. |
|  |  | (G4.4) Ability to redress | Establish mechanisms that allow affected parties to seek recourse or address grievances arising from the AI system's outcomes or actions. |
| Technology | (C5) Technical robustness, safety, and performance | (G5.1) Resilience to attack and security | Develop the AI system to withstand and recover from deliberate attacks and unintended threats, and to maintain consistent and stable behavior in order to ensure the integrity and security of its operation and data. |
|  |  | (G5.2) Contingency management and fallback measures | Conduct risk assessment and implement measures to deal with AI failure, like guardrails or alternative algorithmic or manual controls. |
|  |  | (G5.3) High accuracy and performance | Establish performance measures that align with the goals of the AI system. Perform continuous monitoring and optimization to minimize errors and achieve sufficient and consistent performance. |
|  |  | (G5.4) Reliability and reproducibility | Ensure that the AI provides reliable and reproducible results to avoid harm. This means that AI behaves as expected in a wide range of situations and behaves similarly under the same conditions. |
| HAII | (C6) Human agency and oversight | (G6.1) Ensuring fundamental rights | Guarantee that AI respects fundamental rights of individuals, such as privacy, and non-discrimination. |
|  |  | (G6.2) Human agency | Stakeholders shall retain control and decision-making authority over the AI system and are augmented in their capabilities by it. Stakeholders understand the limitations of the AI system and do not over-rely on it. |
|  |  | (G6.3) Human oversight | Human operators actively monitor and supervise the AI system. |
|  | (C7) Transparency | (G7.1) Traceability | Ensure the ability to trace the development and the reasoning processes behind the AI system. |
|  |  | (G7.2) Explainability | Provide explanations to help the user understand and trust the AI. |
|  |  | (G7.3)  Foster AI awareness, communicate limitations, decision feedback | Ensure the user knows they’re working with an AI. Provide feedback on decisions made by the AI on its own or under the user’s guidance. Inform the user about the strengths and shortcomings of the AI system. Allow the user to provide feedback for improving AI performance. |
|  |  | (G7.4) Intuitive user experience and effective user interface design | Create user-friendly interfaces that seamlessly guide interactions with the AI system, ensuring ease of use, comprehension, and efficient engagement to improve the overall user experience. |
|  | (C8) User adoption and engagement | (G8.1) Education and onboarding | Provide comprehensive and accessible training and orientation materials for users to ensure they have the necessary knowledge and skills to understand and work effectively with the AI system. Ensure a low barrier to start using AI. |
|  |  | (G8.2) User engagement | Implement interactive and motivational elements, such as game-like mechanics and incentives, to encourage sustained user interaction with the AI system. Promote a positive attitude toward AI and a sense of ownership over the AI. |
